# Supplementary material for: Combination venetoclax and selinexor effective in relapsed refractory multiple myeloma with translocation t(11;14)
Source: NPJ Precis Oncol. 2022 Oct 19;6:73. doi: 10.1038/s41698-022-00315-2 (PMC9581939; doi:10.1038/s41698-022-00315-2)
Supplement: Supplementary file 1 — Supplementary Appendix [file 41698_2022_315_MOESM1_ESM.pdf]

Combination Venetoclax and Selinexor Effective in Relapsed Refractory Multiple Myeloma with Translocation t(11;14)

Supplementary Appendix

Table of Contents:

Supplementary Figure and Text (page 2-3)
Supplementary Table (page 4)

Nina Nguyen, MD[1#], Sana Chaudhry, BSc[1#], Tulasigeri M. Totiger, PhD[1#], Robert Diaz, MS[1], Evan Roberts, BSc[1], Skye Montoya, BSc[1], Gabriel Pardo BSc[1], Alejandro Pardo BSc[1], Jumana Afaghani, BSc[1], Maurizio Affer, MSc[1], Jacob Jahn, BSc[1], Terrence Bradley, MD[3], Francesco Maura, MD[2], Dickran Kazandjian, MD[2], Daniel Bilbao, PhD[1], Jennifer Chapman, MD[4], Ola Landgren, MD,PhD[2], James Hoffman, MD[2*], and Justin Taylor, MD[3*]

Supplementary Figure 1

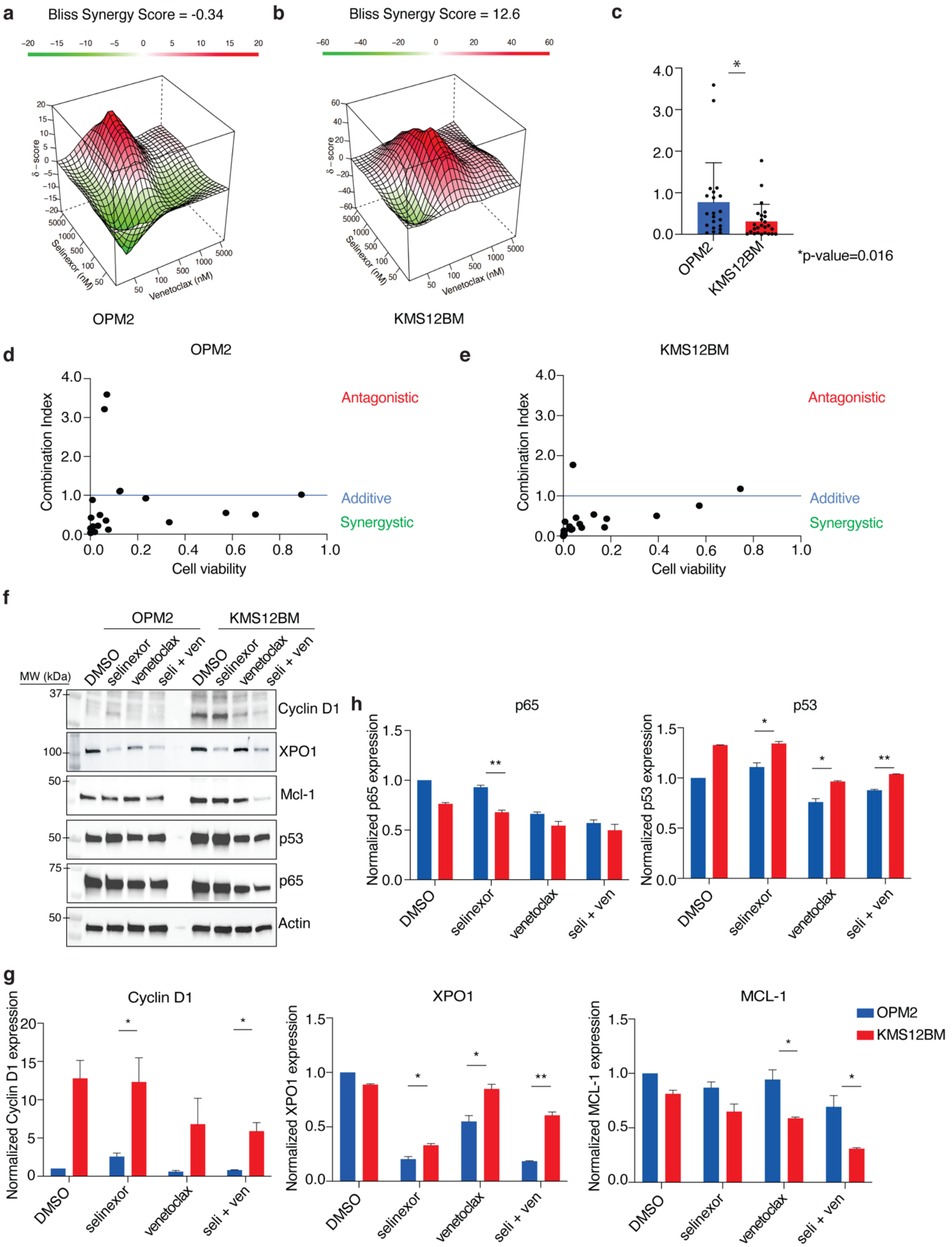

Supplementary Figure 1. The combination of selinexor and venetoclax show higher levels of synergy and decreased levels of Cyclin D1, XPO1, and MCL-1 protein levels in KMS12BM.

OPM2 and KMS12BM were treated with increasing doses of selinexor and venetoclax for 72 hours and cell viability was measured using CellTiter-Glo. Contour plots were calculated using the Bliss Independence model and were generated using the SynergyFinder web application. Red indicates synergism and green indicates antagonism (**a and b**). The synergy of OPM2 and KMS12BM was compared using Combination Index (CI) values. The difference was measured using Student's t-test (**c**). The synergy was also calculated using the CompuSyn software. Combination Index values >1 indicates antagonism, =1 indicates additivity, <1 indicates synergy (**d and e**). OPM2 and KMS12BM were treated with selinexor (200nM) and venetoclax(1μM) for 16 hours and subjected to a Western blot using various antibodies as indicated (**f**). The normalized protein levels of p65, p53, Cyclin D1, XPO1, and MCL-1 was calculated by the intensity of the Western blot bands using Image J software (**g and h**).

Cell Lines	MM IgH translocation
U266-B1	t(11;14)(CCND1/IGH)
KMS-12-BM	t(11;14)(CCND1/IGH)
MOLP-8	t(11;14)(CCND1/IGH)
SK-MM-2	t(11;14)(CCND1/IGH)
NCI-H929	t(4;14)(MMSET/IGH)
LP-1	t(4;14)(MMSET/IGH)
OPM-2	t(4;14)(MMSET/IGH)<br>t(?;20)(?;MAFB)
EJM	t(14;20)(IGH/MAF)
L-363	t(6;20)(?;MAFB)
KARPAS-620	t(8;11)(CCND1)
JJN-3	t(14;16)(IGH/MAF)
MM1S	t(14;16)(IGH/MAF)
KMS-11	t(4;14)(MMSET/IGH)<br>t(14;16)(IGH/MAF)
RPMI-8226	t(16:22)(MAF;IGL)
AMO-1	-
MC-CAR	-
IM-9	-
ARH-77	-

Supplementary Table 1. List of multiple myeloma (MM) cell lines according to their immunoglobulin heavy chain (IgH) translocation.

Abbreviations: CCND1, Cyclin D1; IGH, immunoglobulin heavy chain; MMSET, multiple myeloma SET domain; IGL, immunoglobulin light chain
