# Supplementary figures and images for: Combination venetoclax and selinexor effective in relapsed refractory multiple myeloma with translocation t(11;14)
Source: NPJ Precis Oncol. 2022 Oct 19;6:73. doi: 10.1038/s41698-022-00315-2 (PMC9581939; doi:10.1038/s41698-022-00315-2)

From Figure 2F

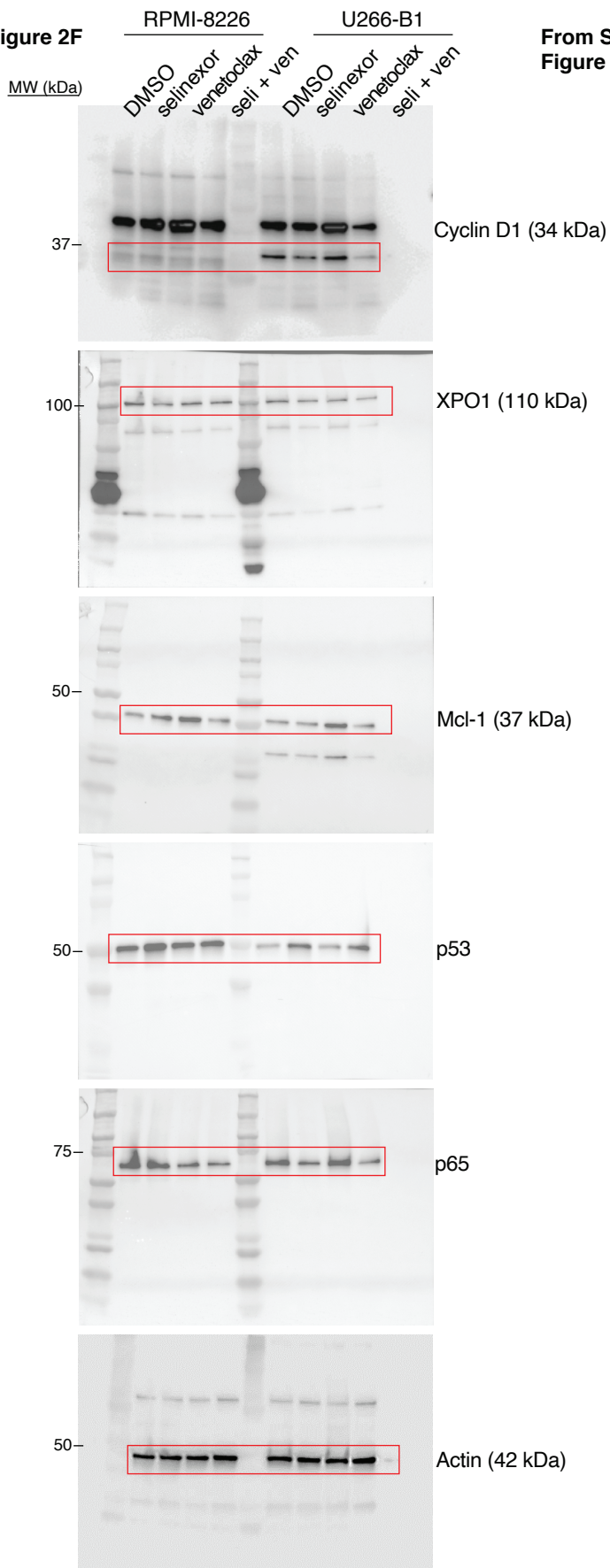

From Supplementary Figure 1F

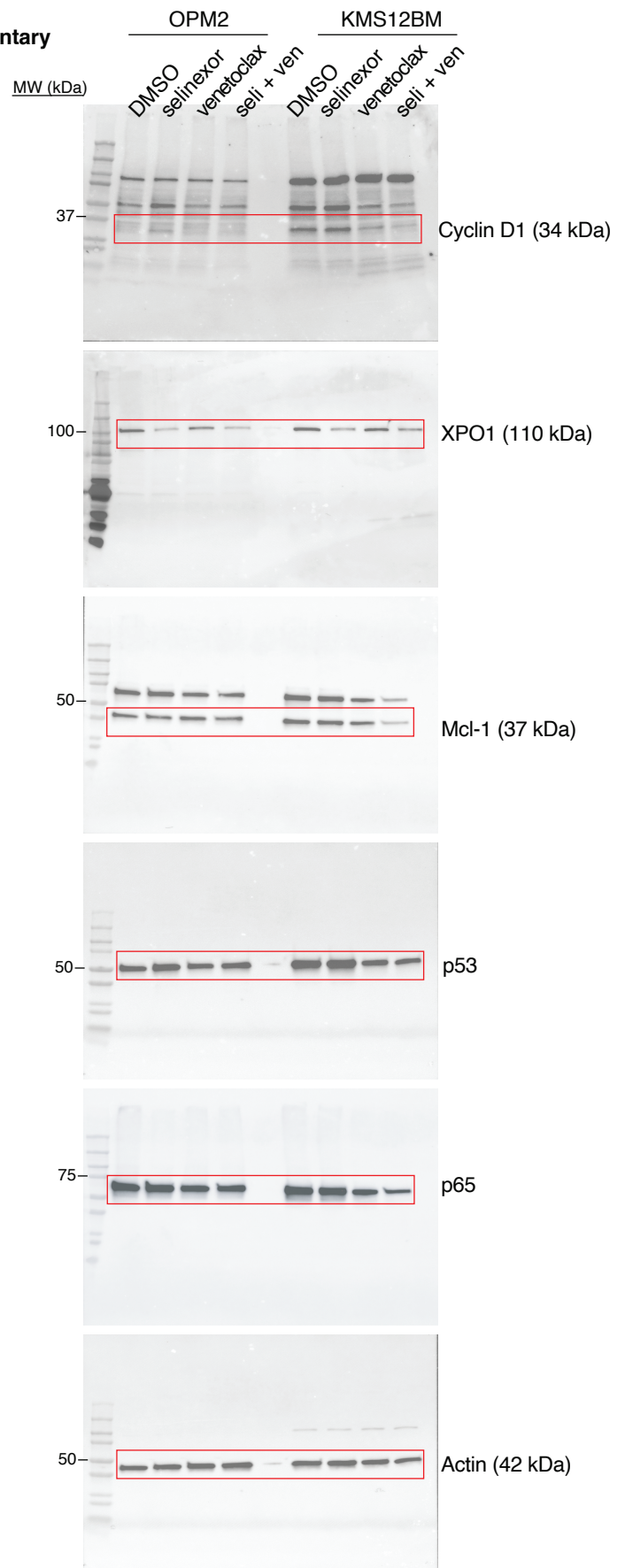

Supplement: Supplementary file 2 — Data Set 1 [file 41698_2022_315_MOESM2_ESM.pdf]
