# Supplementary material for: Combination venetoclax and selinexor effective in relapsed refractory multiple myeloma with translocation t(11;14)
Source: NPJ Precis Oncol. 2022 Oct 19;6:73. doi: 10.1038/s41698-022-00315-2 (PMC9581939; doi:10.1038/s41698-022-00315-2)
Supplement: Supplementary file 3 — REPORTING SUMMARY [file 41698_2022_315_MOESM3_ESM.pdf]

nature portfolio

Corresponding author(s): Justin Taylor

Last updated by author(s): Sep 7, 2022

Reporting Summary

Nature Portfolio wishes to improve the reproducibility of the work that we publish. This form provides structure for consistency and transparency in reporting. For further information on Nature Portfolio policies, see our Editorial Policies and the Editorial Policy Checklist.

Statistics

For all statistical analyses, confirm that the following items are present in the figure legend, table legend, main text, or Methods section.

n/a	Confirmed	
☐	☒	The exact sample size (*n*) for each experimental group/condition, given as a discrete number and unit of measurement
☐	☒	A statement on whether measurements were taken from distinct samples or whether the same sample was measured repeatedly
☐	☒	The statistical test(s) used AND whether they are one- or two-sided<br>*Only common tests should be described solely by name; describe more complex techniques in the Methods section.*
☐	☒	A description of all covariates tested
☐	☒	A description of any assumptions or corrections, such as tests of normality and adjustment for multiple comparisons
☐	☒	A full description of the statistical parameters including central tendency (e.g. means) or other basic estimates (e.g. regression coefficient) AND variation (e.g. standard deviation) or associated estimates of uncertainty (e.g. confidence intervals)
☐	☒	For null hypothesis testing, the test statistic (e.g. *F*, *t*, *r*) with confidence intervals, effect sizes, degrees of freedom and *P* value noted<br>*Give P values as exact values whenever suitable.*
☒	☐	For Bayesian analysis, information on the choice of priors and Markov chain Monte Carlo settings
☒	☐	For hierarchical and complex designs, identification of the appropriate level for tests and full reporting of outcomes
☒	☐	Estimates of effect sizes (e.g. Cohen's *d*, Pearson's *r*), indicating how they were calculated

Our web collection on statistics for biologists contains articles on many of the points above.

Software and code

Policy information about availability of computer code

Data collection	No software was used.
Data analysis	No software was used.

For manuscripts utilizing custom algorithms or software that are central to the research but not yet described in published literature, software must be made available to editors and reviewers. We strongly encourage code deposition in a community repository (e.g. GitHub). See the Nature Portfolio guidelines for submitting code & software for further information.

Data

Policy information about availability of data

All manuscripts must include a data availability statement. This statement should provide the following information, where applicable:

- Accession codes, unique identifiers, or web links for publicly available datasets
- A description of any restrictions on data availability
- For clinical datasets or third party data, please ensure that the statement adheres to our policy

All data will be made available by contacting the corresponding authors.

Human research participants

Policy information about studies involving human research participants and Sex and Gender in Research.

Reporting on sex and gender	Sex and gender analysis was not performed due to the disease equally affecting both sexes.
Population characteristics	Details on the four patients included are described in the manuscript.
Recruitment	Sequential patients were recruited from the clinic.
Ethics oversight	University of Miami IRB

Note that full information on the approval of the study protocol must also be provided in the manuscript.

Field-specific reporting

Please select the one below that is the best fit for your research. If you are not sure, read the appropriate sections before making your selection.

☒ Life sciences ☐ Behavioural & social sciences ☐ Ecological, evolutionary & environmental sciences

For a reference copy of the document with all sections, see nature.com/documents/nr-reporting-summary-flat.pdf

Life sciences study design

All studies must disclose on these points even when the disclosure is negative.

Sample size	No sample size calculation was performed. These were rare patients so all patients available were included.
Data exclusions	No data were excluded
Replication	Experiments were replicated in at least triplicates. All experimental findings were replicated.
Randomization	Randomization was not performed
Blinding	Blinding was not possible.

Reporting for specific materials, systems and methods

We require information from authors about some types of materials, experimental systems and methods used in many studies. Here, indicate whether each material, system or method listed is relevant to your study. If you are not sure if a list item applies to your research, read the appropriate section before selecting a response.

Materials & experimental systems

n/a	Involved in the study	
☐	☒	Antibodies
☐	☒	Eukaryotic cell lines
☒	☐	Palaeontology and archaeology
☐	☒	Animals and other organisms
☐	☒	Clinical data
☒	☐	Dual use research of concern

Methods

n/a	Involved in the study	
☒	☐	ChIP-seq
☒	☐	Flow cytometry
☒	☐	MRI-based neuroimaging

Antibodies

Antibodies used	antibodies against Cyclin D1 (BD Biosciences, San Jose, CA); XPO1 and p53 (Santa Cruz Biotechnology, Dallas, TX); and MCL-1 and p65 (Cell Signaling Technologies, Danvers, MA), all used at 1:1000 dilution in 1% BSA. β-actin (Sigma Aldrich) was used at 1:10,000.
Validation	These were purchased from companies with reputable validation techniques.

Eukaryotic cell lines

Policy information about cell lines and Sex and Gender in Research

Cell line source(s)	RPMI-8226 and U266-B1 cell lines were purchased from ATCC (Manassas, VA). OPM2 and KMS12BM cell lines were a kind gift from Dr. Leif Bergsagel from Mayo Clinic.
Authentication	Cell lines were authenticated by STR sequencing.
Mycoplasma contamination	Cell lines were tested for mycoplasma with the MycoAlert mycoplasma detection kit (Catalog # LT07-218, Lonza, Morristown, NJ).
Commonly misidentified lines (See ICLAC register)	N/A

Animals and other research organisms

Policy information about studies involving animals; ARRIVE guidelines recommended for reporting animal research, and Sex and Gender in Research

Laboratory animals	Mice, NSG-SGM3, Female, Age 3 months
Wild animals	N/A
Reporting on sex	Sex and gender analysis was not performed due to the disease equally affecting both sexes.
Field-collected samples	N/A
Ethics oversight	University of Miami has full AAALAC accreditation; complies with Federal laws and regulations including the Animal Welfare Act (AWA) regulations overseen by the USDA, PHS Policy on Humane Care and Use of Laboratory Animals administered by the NIH, Office of Laboratory Animal Welfare (OLAW); and has an assurance letter on file with the DHHS (assurance #A3224-01).

Note that full information on the approval of the study protocol must also be provided in the manuscript.

Clinical data

Policy information about clinical studies
All manuscripts should comply with the ICMJE guidelines for publication of clinical research and a completed CONSORT checklist must be included with all submissions.

Clinical trial registration	N/A
Study protocol	Protocol is on file with IRB
Data collection	Data were collected by retrospective chart review
Outcomes	Outcomes were not on a clinical trial and just for the four patients included.
